# The influence of antibiotics on the anammox process — a review

**DOI:** 10.1007/s11356-021-17733-7

**Published:** 2021-11-29

**Authors:** Filip Gamoń, Grzegorz Cema, Aleksandra Ziembińska-Buczyńska

**Affiliations:** Environmental Biotechnology Department, Silesian University of Technology, Akademicka 2A, 44-100 Gliwice, Poland

**Keywords:** Antibiotics, Anammox, Process activity, Wastewater pollutants, Antibiotic resistance mechanisms

## Abstract

Anaerobic ammonium oxidation (anammox) is one of the most promising processes for the treatment of ammonium-rich wastewater. It is more effective, cheaper, and more environmentally friendly than the conventional process currently in use for nitrogen removal. Unfortunately, anammox bacteria are sensitive to various substances, including heavy metals and organic matter commonly found in the wastewater treatment plants (WWTPs). Of these deleterious substances, antibiotics are recognized to be important. For decades, the increasing consumption of antibiotics has led to the increased occurrence of antibiotics in the aquatic environment, including wastewater. One of the most important issues related to antibiotic pollution is the generation and transfer of antibiotic resistance bacteria (ARB) and antibiotic resistance genes (ARGs). Here, we will discuss the effect of short- and long-term exposure of the anammox process to antibiotic pollutants; with a special focus on the activity of the anammox bacteria, biomass properties, community structures, the presence of antibiotic resistance genes and combined effect of antibiotics with other substances commonly found in wastewater. Further, the defense mechanisms according to which bacteria adapt against antibiotic stress are speculated upon. This review aims to facilitate a better understanding of the influence of antibiotics and other co-pollutants on the anammox process and to highlight future avenues of research to target gaps in the knowledge.

## Introduction

The anaerobic ammonium oxidation process (anammox) has been used in the treatment of ammonium-rich wastewater with organic carbon deficiency, where ammonium is oxidized by anammox bacteria to nitrogen gas using nitrite as electron acceptor (Strous et al. 1998). Additionally, the anammox process for the treatment of wastewater provides important benefits compared to widespread nitrification and denitrification process such as the reduction of oxygen demand (by ca. 60%), the elimination for the addition of external carbon sources (which caused a decrease in operational cost (up to 60%)), low excess sludge production due to long doubling time (9–29 days), a high nitrogen removal rate, and a lower greenhouse gases emission (by ca. 90%) (Tomaszewski et al. 2017). While superior to older technologies, the anammox process is sensitive to changes in environmental and chemical parameters such as temperature, pH, and nitrogen load. Further, the anammox process is also vulnerable to a wide range of inhibiting substances, such as antibiotics, heavy metals, free ammonia, free nitrous acid, nitrite, salts, organic matter, phosphate, sulfides, and 1,4-dioxane (Tomaszewski et al. 2017, Jin et al. 2012, Lotti et al. 2012a, Ismail et al. 2021a, b). This sensitivity makes the anammox process impossible to be implemented for treatment in most types of wastewater due to the excessive number of pollutants which are found in these aqueous environments (Shi et al. 2017).

More and more scientific attention is being given to antibiotics as micropollutants in the environment. Globally, antibiotics are widely used in human and veterinary medicine, both for treating bacterial infection and for prophylaxis (Kümmerer 2009; Jin et al. 2012). High concentrations of antibiotics have been detected in a diverse range of aquatic environments including bank filtrates, wastewater, surface water and even groundwater (Kümmerer 2009, Watkinson et al. 2009; Yang et al. 2013). The most common antibiotics detected in these environments include amoxycillin (AMX), penicillin G, norfloxacin (NOR), enrofloxacin (ENR), ciprofloxacin (CIP), erythromycin (ERY), tetracycline (TC), oxytetracycline (OTC), and sulfamethoxazole (SMX) (Watkinson et al. 2009). The occurrence of antibiotics in the environment is a serious problem for living organisms due to the adverse effects these compounds have on their physiology. Additionally, antibiotics are designed to exert specific biological activity and, in some species, may have an immediate effect on bacterial homeostasis (Loos et al. 2013). Moreover, long-term exposure to even sub-inhibitory concentrations of antibiotics may cause chronic toxicity (Bengtsson-Palme and Larsson 2016).

Antibiotics and their metabolized products can induce unforeseen changes in an individual bacterium which can then become common in bacterial communities (Grenni et al. 2018). For example, the occurrence of antibiotics in the aquatic environment has been linked to the development of antibiotic resistance genes (ARGs) and antibiotic-resistant bacteria (ARB) (Gonzalez Ronquillo and Angeles Hernandez 2017; Kumar et al. 2019; Agrawal et al. 2020). ARGs are located on the chromosome or embedded in mobile genetic elements. Further, their location on the mobile genome elements, such as plasmids or transposons, determines their mobility among microorganisms. Additionally, WWTPs have been identified as important reservoirs of antibiotic resistance genes, due to the high density of microorganisms and high concentration of organic substances which can facilitate the transfer of antibiotic resistance genes (Osińska et al. 2019).

Previous studies have shown that anammox activity is inhibited by antibiotics (van de Graaf et al. 1995, Fernández et al. 2009, Yang et al. 2013, Shi et al. 2017). However, it was reported that the anammox process and partial nitrification were successfully performed in the treatment of piggery wastewater which was reported to include a high concentration of antibiotics (Suto et al. 2017). Moreover, the anammox process is used for landfill leachate treatment as well as livestock and swine wastewater and pharmaceutical wastewater (Jin et al. 2012; Tang et al. 2011), where antibiotics were also found. For instance, it was presented that concentration of OTC can reach a few mg L^−1^ in livestock wastewater, because of the broad administration of these antibiotics in livestock, while concentration of antibiotics in pharmaceutical wastewater reach a few µg L^−1^ (Felis et al. 2019). Despite the application of the anammox process to the treatment of pharmaceutical wastewater (for example Jin et al. 2012 Tang et al. 2011), the authors suggested that the conventional anammox process is not suitable for this kind of wastewater because of cumulative toxicity. Moreover, antibiotics can variably affect the anammox process leading to the inhibition of nitrogen removal efficiency, altering the structure of microbial communities and stimulating the transfer of ARGs (Rodriguez-Sanchez et al. 2017; Du et al. 2018; Zhang et al. 2019b). Moreover, previous studies have reported that some antibiotics can be adsorbed or metabolized by anammox bacteria (Zhang et al. 2014). However, the exact mechanism by which antibiotics influence the anammox process remains unclear.

Here, we review the current studies and literature which interrogate the short- and long-term influence of antibiotic exposure on the anammox process. Further, we plan to link these data to bacterial activity, activated sludge granule properties, microbial community structure and the combined effect of antibiotics and other contaminants in wastewater. Additionally, we will also discuss the way of future study which will allow for more comprehensive understanding the impact of antibiotics on the anammox process. From the point of the implementation of anammox process, this review will provide the information about the possible problems that might have occurred in the anammox system in the WWTPs as a result of the presence of antibiotics in wastewater. Moreover, we would like to point out the directions in which further research on the influence of antibiotics on biological nitrogen removal processes, especial Anammox, should follow.

## Effect of antibiotics on the anammox process

### Short-term exposure effect of antibiotics on anammox process

Current research into the effect of antibiotics on the anammox process has primarily focused on both the short- and long-term exposure of antibiotics. However, both, short- and long-term should be specifically distinguished due to the various abilities of bacteria to adapt at these two timepoints (Du et al. 2018; Zhang et al. 2019b). Thus, a comprehensive data set describing the biological changes which may occur in anammox bacteria in response to short-term antibiotic exposure is urgently required. Table 1 reports the short-term effects of different concentrations of antibiotics on anammox process. From this table, it is clear that the most investigated antibiotic assessed during short-term antibiotic exposure on the anammox process is chloramphenicol (CAP). CAP is widely used for animal breeding (Phanwilai et al. 2020). Further, it has been shown that residual CAP can leach into the aquatic environment from the animal excrement (Díaz-Cruz et al. 2003; Phanwilai et al. 2020). Moreover, it has been demonstrated that CAP affected most bacteria in WWTPs (Dapena-Mora et al. 2007; Fernández et al. 2009; Phanwilai et al. 2020) and also the human hemopoietic system (Festing et al. 2001). Van der Graff reported that CAP, at a concentration of 200 mg L^−1^_,_ caused the inhibition of anammox activity by 95% (van der Graff et al. 1995). A similar finding was reported by Fernández et al. (2009) and Phanwilai et al. (2020). Both studies provided evidence that CAP could significantly affect anammox activity. However, there were several experimental differences in studies mentioned. Namely, Fernández et al. (2009) used higher concentrations of CAP (250, 500, 1000 mg L^−1^) in comparison to Phanwilai et al. (2020) (5, 10, 20, 50, 100 mg L^−1^). Moreover, Phanwilai et al. (2020) used two types of biomass (suspended- and attached-growth), while Fernández et al. (2009) investigated only granular sludge. Additionally, Phanwilai et al. (2020) assessed the effect of CAP at an earlier exposure time (7 h), relative to Fernández et al. (2009) (24 h). In contrast to these studies, an entirely different result for 1000 mg L^−1^ CAP was obtained by Dapena-Mora et al. (2007), where no inhibitory effect was observed at 5–6 h. It is important to note, however, that Dapena-Mora et al. (2007) used flock biomass enriched in bacteria belonging to *Kuenenia stuttgartiensis.* Van de Graaf et al. (1996) indicated that the degree to which CAP can affect anammox enrichment is linked to the microbial parameters of the culture being assessed. Hu et al. (2013) elucidated the effect of penicillin G and streptomycin at concentrations ranging from 0–2000 mg L^−1^ to 0–200 mg L^−1^, respectively. Their data indicated that a microbial biomass, with low biodiversity, is more resistant to antibiotic stress during short-term exposure. Therefore, due to the fact that anammox bacteria co-exist with other nitrogen cycle bacteria in the anammox biomass (Ziembińska-Buczyńska et al. 2019; Banach-Wiśniewska et al. 2019), it can be assumed that the high diversity of these microorganisms will influence the activity of anammox biomass.

**Table 1:** Summary of short-term effect of antibiotics on AnAOB

Antibiotic	Concentration	Biomass	Activity	Exposure time	References
Chloramphenicol	200 mg L^−1^	Granules	Decreased by 95%	First 3 days of incubation	Van der Graaf et al. (1995)
	250–1000 mg L^−1^	Granular	Decreased by 20–80%	24 h	Fernández et al. 2009
	100–1000 µg L^−1^	Granular	No effect	24 h	Fernández et al. 2009
	1000 mg L^−1^	Granular, Anammox biomass enriched in bacteria belonging to the specie Candidatus *Kuenenia stuttgartiensis*	No effect	No information	Dapena-Mora et al. (2007)
	5–100 mg L^−1^	Biofilm	Decreased by 16.2–44.7%	7 h	Phanwilai et al, (2020)
	5–100 mg L^−1^	Suspended-growth	Decreased by 29.9–45.5%	5 h	Phanwilai et al. (2020)
	100–1000 µg L^−1^	Biofilm	No effect	24 h	Phanwilai et al. (2020)
	100–1000 µg L^−1^	Suspended-growth	No effect	24 h	Phanwilai et al. (2020)
Tetracycline hydrochloride	100–1000 mg L^−1^	Granular	Decreased by 20–80%	24 h	Fernández et al. 2009
Oxytetracycline	25–100 mg L^−1^	Granular	TNRR decreased from 74.73% to 33.45–22.15%	7 h	Noophan et al. (2012)
Doxycycline	50–100 mg L^−1^	Granular	Decreased by 14–17%	24 h	Sguanci et al, (2017)
	100 mg L^−1^	Granular	Decreased by 47.6%	24 h	Alvarino et al. (2014)
	50–100 mg L^−1^	Granular	Decreased by 22%	48 h	Sguanci et al. (2017)
Tiamulin	50 mg L^−1^	Granular	Decreased by 5%	24 h	Sguanci et al. (2017)
	50–500 mg L^−1^	Granular	Decreased by 5–64%	48 h	Sguanci et al. (2017)
Enrofloxacin	50 mg L^−1^	Granular	Decreased by 13%	24 h	Sguanci et al. (2017)
	100–200 mg L^−1^	Granular	Decreased by 42–60%	48 h	Sguanci et al. (2017)
Penicilin G	0–2000 mg L^−1^	Flocs, *Kuenenia stuttgartiensis* (free-living planktonic cells)	No effect	1 h	Hu et al. (2013)
Streptomycin	0–200 mg L^−1^	Flocs, *Kuenenia stuttgartiensis* (free-living planktonic cells)	No effect	1 h	Hu et al. (2013)
Norfloxacin	1 µg L^−1^*	Biofilm	Decreased by 2.2%	6 h	Zhang et al. (2019a)
Erythromycin	1 µg L^−1^*	Biofilm	decreased by 0.56%	6 h	Zhang et al. (2019a)

*TNRR*, total nitrogen removal rate. * Studies using the concentration of antibiotics that occur in real wastewater

One of the antibiotics group most often used in medicine and animal breeding are tetracyclines (TCs). TCs inhibit the biosynthesis of proteins by preventing the binding of aminoacyl-tRNA to the 30S ribosome subunit (Yang et al. 2005). Noophan et al. (2012) investigated the effect of oxytetracycline at concentrations ranging from 23 to 100 mg L^−1^. In that study, the authors reported a partial reduction of the anammox activity. Further, Sguanci et al. (2017) compared the effect of three antibiotics: doxycycline (DOX), tiamulin (TIA) and enrofloxacin (ENR) after 24 and 48 h. From their data, they reported that the inhibition of the anammox process was recorded after 48-h exposure to 50 mg L^−1^ of the following compounds in the order of DOX > ENR > TIA.

The major problem with the current studies is the level of antibiotics concentrations used. Most of the studies presented the results of the experiments with the antibiotics concentration that does not occur in real wastewater; therefore, it is hard to evaluate the real impact of antibiotics on the anammox process implemented in full-scale reactor. A few studies have investigated the effect of trace concentrations (< 1 mg L^−1^) of antibiotics on the anammox bacteria. However, current evidence suggests that concentrations less than 1 mg L^−1^ do not affect the anammox bacteria (Fernández et al. 2009; Phanwilai et al. 2020). In contrast to these data, Zhang et al. (2019a) reported the effect on anammox bacteria to a low concentration of NOR and ERY (< 1 µg L^−1^). In this study, the authors found that specific anammox activity (SAA) slightly decreased from 10.8 mg g^−1^ SS h^−1^ for both NOR and ERY to 10.56 and 10.74 mg g^−1^ SS h^−1^, respectively. Despite the low impact of ERY and NOR on the SAA, it was further reported that dehydrogenase activity (DHA) under NOR exposure was significantly suppressed in the short-term test where it decreased from 0.49 to 0. 39 EU g^−1^ SS.

Based on short-term exposure, the half-maximal inhibitory concentration (IC_50_) and half-maximal effective concentration (EC_50_) value for antibiotics was designated by some authors. The IC_50_ and EC_50_ values of chosen antibiotics are shown in Table 2. However, different values are reported by the authors for the same antibiotics, especially OTC. This phenomenon may be due to the diversity of the biomass used or the test length as well as exposure time and concentration of the antibiotic (Sguanci et al. 2017). Moreover, it is worth to notice, that all presented EC_50_ and IC_50_ values (Table 2) are much higher than concentration of antibiotics that occur in real wastewater. For instance, the OTC concentration in manure samples have reached even the concentration of 250 mg L^−1^ (Zhang et al. 2014) which is also lower than IC_50_ value presented for this antibiotic. Therefore, there is no possibility to observe acute toxicity of the anammox bacteria used for real wastewater treatment.

Most of the antibiotics used show a negative dose-dependent effect on the anammox process during short-term tests. However, microbial biomass with low biodiversity was found to be more resistant to antibiotics. For antibiotics such as penicillin G and streptomycin, the long doubling time of the anammox bacteria makes it impossible to determinate its inhibition in such a short incubation time (Hu et al. 2013). Thus, to test the effect of these antibiotics, a longer experiment is required. Moreover, in most cases, antibiotic concentrations used in short-term tests were similar to those showing acute effect; therefore, it is difficult to assess short-term effect of antibiotics in practice, because such high concentrations do not occur in real wastewater.

**Table 2:** EC_50_ and IC_50_ values of antibiotics for anammox bacteria

Antibiotics	EC_50_ value	IC_50_ value	Exposure time	References
Chloramphenicol	420 mg L^−1^	-	5 min	Fernández et al. 2009
	390 mg L^−1^	-	15 min	Fernández et al. 2009
Tetracycline hydrochloride	94 mg L^−1^	-	5 min	Fernández et al. 2009
	42 mg L^−1^	-	15 min	Fernández et al. 2009
Oxytetracycline	-	517.5 mg L^−1^	-	Yang et al. (2013)
	-	518 mg L^−1^	-	Zhang et al. (2014)
	-	1100 mg L^−1^	24 h	Lotti et al. (2012b)
Doxycycline	-	121 mg L^−1^	24 h	Alvarino et al. (2014)
	-	665 mg L^−1^	24 h	Sguanci et al. (2017)
	-	378 mg L^−1^	48 h	Sguanci et al. (2017)
Tiamulin	-	920 mg L^−1^	24 h	Sguanci et al. (2017)
	-	371 mg L^−1^	48 h	Sguanci et al. (2017)
Enrofloxacin	-	157 mg L^−1^	24 h	Sguanci et al. (2017)
	-	144 mg L^−1^	48 h	Sguanci et al. (2017)
Sulfathiazole	-	650 mg L^−1^	24 h	Lotti et al. (2012b)

### Long-term exposure effects

Due to fact that anammox bacteria have long double times (9–29 days) as well as a slow growth rate (0.0033–0.001 d^−1^) (Van de Graaf et al. 1996; Strous et al. 1998, 1999), the response of anammox bacteria to antibiotics may be longer. For these reasons, long-term exposure must be investigated. Table 3 reports on the long-term effect of different concentrations of antibiotics on the anammox process.

**Table 3:** Summary of long-term effect of antibiotics on AnAOB

Antibiotic	Concentration	Reactor	Biomass	Effect	Operating time	References
Chloramphenicol	200 mg L^−1^	FBR	Granules	Anammox process activity decreased by 68%	After 3 days	Van der Graaf et al. (1995)
	20 mg L^−1^	FBR	Granules	Anammox process activity decreased by 36%	After 3 days	Van der Graaf et al. (1995)
	20 mg L^−1^	SBR	Granules	Anammox process activity decreased by 25%	22 days	Fernández et al. 2009
	6 mg L^−1^	SBR	Granules	SAA decreased by 86%	41 days	Phanwilai et al. (2020)
	6 mg L^−1^	SBR	Flocs	SAA decreased by 82%	27 days	Phanwilai et al. (2020)
	1000 µg L^−1^	SBR	Granules	SAA increased from 0.43 to 0.46 g N g^−1^ VSS d^−1^	14 days	Phanwilai et al. (2020)
	1000 µg L^−1^	SBR	Flocs	SAA decreased from 0.63 to 0.57 g N g^−1^ VSS d^−1^	14 days	Phanwilai et al. (2020)
Tetracycline hydrochloride	10 mg L^−1^	SBR	Granules	Anammox process activity decreased by 60%	37 days	Fernández et al. 2009
Oxytetracycline	50 mg L^−1^	UASB	Granules	NRR decreased by 4.5 kg N m^−3^ d^−1^	7 days	Yang et al. (2013)
	5 ± 3.5 mg L^−1^	SBR	Granules	Complete inactivation of the anammox process	35 days	Noophan et al. (2012)
	1 mg L^−1^	UASB	Granules	TNRE decreased from 88 to 62.4 ± 12.5% NRR decreased from 20 to 14.3 kg N m^−3^d^−1^	27 days	Zhang et al. (2019c)
	1–2 mg L^−1^	UASB	Granules	TNRE decreased from 92.0 to 50.3% NRR decreased from to 3.6 ± 1.0 kg N m^−3^ d^−1^	20 days	Shi et al. (2017)
	2 mg L^−1^		Granules	NRR decreased to 60.0%	120 days	Zhang et al. (2018a, b)
Penicillin G	100 mg L^−1^	FBR	Granules	Anammox process activity decreased by 36%	After 3 days	Van der Graaf et al. (1995)
	500–5000 mg L^−1^	CMR	Flocs, Candidatus *Kuenenia stuttgartiensis* (free-living planktonic cells)	Completely inactivate of anammox process	21 days	Hu et al. (2013)
Ampicillin	800 mg L^−1^	FBR	Granules	Anammox process activity decreased by 94%	After 3 days	Van der Graaf et al. (1995)
Streptomycin	100 mg L^−1^	CMR	Flocs, *Kuenenia stuttgartiensis* (free-living planktonic cells)	Completely inactivate of anammox process, washout of anammox biomass and increase nitrite concentration in effluent from 0 to 5 mM	17 days	Hu et al. (2013)
Norfloxacin	1 µg L^−1^*	Cylindrical biofilter	Biofilm	SAA decreased from 10.8 to 7.56 mg g^−1^ SS h^−1^	30 days	Zhang et al. (2019a)
Erythromycin	1 µg L^−1^*	Cylindrical biofilter	Biofilm	SAA decreased from 10.8 to 10.65 mg g^−1^ SS h^−1^	30 days	Zhang et al. (2019a)
Erythromycin	0.001*; 1; 10; 50 mg L^−1^	UAF	Biomass enriched with Candidatus *Kuenenia* sp.	NRR decreased from 0.34 kg N m^−3^ d^−1^ to 0.3, 0.274, 0.2, 0.17 g N m^−3^ d^−1^, respectively	119 days	Zhang et al. (2019b)
Sulfamethoxazole	1 mg L^−1^	UASB	Granules	TNRE decreased from 88 to 57.3 ± 11.5% NRR decreased form 20 to 13.2 ± 2.7 kg N m^−3^d^−1^	27 days	Zhang et al. (2019c)
Oxytetracycline + Sulfamethoxazole	1 + 1 mg L^−1^	UASB	Granule	TNRE decreased from 88 to 68.6 ± 10.7% NRR decreased from 20 to 15.2 ± 1.9 kg N m^−3^d^−1^	27 days	Zhang et al. (2019c)

*CMR*, Continuous membrane reactor; *FBR*, fluidized bed reactor; *NRR*, nitrogen removal rate; *SAA*, specific anammox activity, *SBR*, sequential batch reactor; *SS*, suspended solid; *TNRE*, total nitrogen removal efficiency; *UAF*, up-flow anaerobic biological filter; *UASB*, up-flow anaerobic sludge blanket digestion; *VSS*, volatile suspended solid. * Studies using the concentration of antibiotics that occur in real wastewater

Phanwilai et al. (2020) compared the effect of CAP on granular and flock anammox biomass. In that study, the authors reported that the impact of 6 mg L^−1^ CAP on both types of biomass was similar. Further, their data indicates that the SAA decreased by 86% and 82%, respectively. However, when the concentration was increased to 1000 µg L^−1^, a slight increase in SAA was observed. The authors concluded that a granular system is more resistant to antibiotic inhibition relative to a flock system. Therefore, anammox granules seem to be preferred for use in antibiotic-contaminated wastewater treatment. This is primarily because of the multi-layered structures with two distinct regions — dispersible and stability layers. The outer layer is dispersible because the particles adhere to each other through weak interaction, i.e., extracellular polymeric substances (EPSs) ion bridging via multivalent ions and van der Waals forces. The inner region, composed of compact EPSs, is stable and the particles interact strongly through polymer entanglement (Zhang et al. 2015). However, the concentration of CAP in the effluent of anaerobic treatment process could be higher than in the effluent of WWTPs, where conventional systems (nitrification/denitrification) are used, because CAP would not bio-degrade under anaerobic processes (Michael et al. 2013).

Yang et al. (2013) indicated that the IC_50_ value of OTC is 517.5 mg L^−1^ for the anammox process, however, a concentration of only 50 mg L^−1^ can deteriorate the nitrogen removal ability of anammox bacteria within 7 days. Furthermore, 5 ± 3.5 mg L^−1^ of OTC completely inhibited the anammox process by 5 weeks (Noophan et al. 2012). An equally strong effect on anammox bacteria was caused by TCH (Fernández et al. 2009). From these data, Fernández et al. (2009) suggested that the application of the anammox process to pharmaceutical wastewater treatment requires the simultaneous dual-usage of a degradation technique, which should mitigate the negative effect of these biodegradable-resistant antibiotics. Such methods may include photo-degradation and chlorination (Addamo et al. 2005; Qiang et al. 2006). It was found that anammox bacteria can adapt to the low concentration of SM and SDM (< 3 mg L^−1^) when incubated in an up-flow anaerobic sludge blanket (UASB) reactor, because of increasing EPSs production as a defense mechanism. Further, the inhibition and accumulation of ammonium at 9 mg L^−1^ of SM and SMD were observed. However, SM in concentrations between 5 and 9 mg L^−1^ was more inhibitory than SDM due to the decrease in the use of nitrite by anammox bacteria, which led to the accumulation of nitrite (Du et al. 2018).

In contrast to the short-term effect, trace concentrations (1 µg L^−1^) of NOR and ERY for 30 days was reported to decrease anammox activity by 30% and 1.4%, respectively (Zhang et al. 2019a). These results highlight the stronger inhibitory action of NOR relative to ERY for the suppression of the anammox bacterial. However, the action of ERY seems to be different for each type of wastewater treatment bacteria. For example, the addition of 1 mg L^−1^ of ERY slightly inhibited nitrite-oxidizing bacteria (NOB) during long-term exposure (Du et al. 2016), and dramatically decreases phosphorus removal process within 7 days (Hu et al. 2018a, b). A comparison of the action of the three broad-spectrum antibiotics: mainly amoxicillin (AMX), florfenicol (FF), sulfamethazine (SMX) was studied by Zhang et al. (2015). Long-term testing in the UASB has shown that the significant inhibition of the SAA was observed when the concentration of antibiotics was 1000, 230, 128 mg L^−1^ for AMX, SMZ, FF, respectively. Moreover, the SAA decreased by approximately 50% within the first 3 days.

Similar to the short-term exposure studies, the long-term exposure studies mainly used high concentrations of antibiotics that do not occur in real wastewater. There are only a few studies that use concentrations of antibiotics found in the aquatic environment (few µg L^−1^) including wastewater. Zhang et al. (2019a, b) used ERY and NOR to investigate their effect on the anammox process under 1 µg L^−1^. In both studies, no inhibitory effect of such low concentrations of antibiotics on the anammox process was demonstrated. Furthermore, the results presented in Table 3, obtained in these works, should not be taken as a real decrease in the activity of the anammox process, since there is a large scatter in the results when based on activated sludge. Therefore, such slight changes in the activity of the anammox process should not be considered as a direct effect of the antibiotics influence on the process.

Antibiotics co-occur in aquatic environments. Thus, it is necessary to study their joint effect on the anammox systems. To assess this, the individual and combined effect of OTC and SMX was investigated by Zhang et al. (2019c). Their results showed that the anammox process deteriorated when the concentration of both antibiotics reached 1 mg L^−1^. However, the impact of separate antibiotics on anammox was higher than the combined groups.

Long-term effect of antibiotics should be connected with sludge retention time (SRT) that is used in experiment. For the anammox process, SRT should range from 30 to 60 days or even more (Chowdhury and Nakhla 2021). In the results presented in Table 3, it can be seen that, in most cases, the exposure time is lower than the optimal SRT for the anammox process mentioned above. The use of too short SRT may lead to a decrease in the efficiency of the anammox process, which may be important for studies involving the effects of antibiotics (Chowdhury and Nakhla 2021).

To sum up, the differences between long- and short-term exposure are mainly due to the characteristics of anammox bacteria, which have a low growth rate. Thus, a short incubation period may be too short to characterize the effect mediated by antibiotic pollution (Hu et al. 2013). Additionally, the production of EPSs seems to be a self-protection mechanism of bacteria against antibiotics during short-term tests. Antibiotics could be adsorbed by the EPSs, reducing the short-time risk of direct contact of antibiotics and anammox bacteria (Xu et al. 2013; Zhang et al. 2015). Moreover, the inhibitory concentration of antibiotics is highly dependent on the different types of antibiotics. For example, tetracycline and sulfonamides present more significant disturbing in the anammox process than chloramphenicol or penicillin. In addition, the level of inhibition might also be attributed to the community structures, type of biological reactor/technology, and operational conditions.

### Effect of antibiotics on anammox functional genes

The relative abundance of certain functional genes may be a useful tool to assess the efficiency of biological processes mediated by microorganisms (Zhi and Ji 2014). In the anammox metabolic pathways, there are three functional genes which are recognized to be the most important: nitrite reductase (*nir)* which catalyzes the conversion of nitrite into nitric oxide (Li et al. 2011), hydrazine synthase (*hzs*) which induces the transformation of nitric oxide into hydrazine, and hydrazine dehydrogenase (*hdh*) which covers hydrazine to nitrogen gas (Kartal et al. 2011).

It has been reported that SMD in concentrations ranging between 1 and 5 mg L^−1^ did not significantly affect the absolute abundance of *hzsA*. However, when the concentration reached 7 mg L^−1^, the abundance dropped significantly from 1.1 × 10^6^ to 3.0 × 10^5^ copies/ng DNA. Further, at 1 mg L^−1^ of SMD, the abundance of *hzo* was markedly increased from 8.0 × 10^6^ to 3.3 × 10^7^ and next decreased at 7 mg L^−1^. Similarly, SD at 1 mg L^−1^ caused a significant increase in *hzs* abundance. These data indicate that anammox bacteria are more sensitive to SMD (Du et al. 2018). A low concentration of SMX (0.1 mg L^−1^) stimulated the expression of *nirS*, *hzsA* and *hdh* mRNA. Additionally, when the concentrations of OTC and SMX increased from 0.1 to 0.5 mg L^−1^, the sensitivity of *nirS*, *hzsA* and *hdh* to these antibiotics was as follows: SMX > OTC > SMC + OTC. Thus, the response of *hzsA* to OTC at 1 mg L^−1^ is higher than SMX and SMX + OTX. On the other hand, *hdh* was the most sensitive to 0.1 mg L^−1^ OTC (Zhang et al. 2019c). Zhang et al. (2019c) demonstrated that within anammox bacteria, 2 mg L^−1^ of OTC was able to inhibit the *hzsA*, *hdh*, *nirS* genes at the mRNA level. The combined effect of Zn (II) and TC on the *hzsA*, *hdh*, *nirS* expression level was studied by Fan et al. (2019). The authors found a similar trend in the relative abundance of these genes. When the concentration of Zn (II) and TC was 3.39 and 0.5 mg L^−1^, respectively, the relative abundance was slightly increased. At higher concentrations of antibiotics (1.0 mg L^−1^), the relative abundance of the functional genes gradually dropped below 0.1% and was significantly lower than that of the control reactor. Taken together, these lines of evidence highlight the impact of antibiotics on the expression of functional genes within the anammox process.

### Antibiotic influence on the anammox microbial community

Current papers have shown that dominant phyla in the anammox system are *Planctomycetes*, *Proteobacteria* and *Firmicutes*, however, the quantity of *Firmicutes* was significantly lower than other phyla (Zhang et al. 2019b, c). However, physical and chemical parameters within these anammox systems strongly influence functional bacteria group responsible for the process. It has been reported in the literature that *Proteobacteria* include antibiotic-resistant bacteria (Shi et al. 2013). Further, Duan et al. (2017) also reported that *Proteobacteria* can use OTC as a carbon source. Similarly, *Betaproteobacteria* have a high antibiotic resistance genes exchange capability, for this reason, they are the dominant bacteria found in the OTC-rich wastewater (Liu et al. 2012). Additionally, the groups of bacteria with tetracycline resistance genes also include the *Firmicutes* phylum (Ghosh and LaPara 2007). Zhu et al. (2017) stated that *Gammaproteobacteria* have a good ability to obtain ARGs under high antibiotic concentration conditions.

Studies have reported that OTC at a concentration of 5 ± 3.5 mg L^−1^ induced significant changes in anammox community structure during a 5-week experiment. Further, it was found that Candidatus *Brocadia anammoxidans* and Candidatus *Kuenenia stuttgarteinsis* had a lower density at the 5-week timepoint as compared to the beginning of the experiment. However, higher numbers of *Nitrosomonas* sp. and *Nitrospira* sp. were observed (Noophan et al. 2012). From these data, the authors concluded that as nitrifying bacteria can survive at a concentration of 250 mg L^−1^ with only a 50% reduction in activity (Campos et al. 2001), they have a higher tolerance to OTC than anammox bacteria. Zhang et al. (2019c) found that Candidatus *Kuenenia* sp. gradually adapted to the OTC and SMX exposure with a relative abundance of over 31%. These data illustrate that the long-term adaptation to OTC and SMX may improve the tolerance to these antibiotics. However, the combined effect of these two antibiotics was found to be more severe for Candidatus *Kuenenia* sp. causing a significant decrease in their abundance which was proportional to the exposure time to the antibiotics. Nonetheless, *Candidatus Kuenenia* sp. is considered to be potentially an antibiotic-resistant bacterium (Zhang et al. 2018a, b). The combined effect of OTC and copper nanoparticles on the anammox bacterial community was shown in *Planctomycetes.* In contrast to the findings in *Proteobacteria*, the authors reported the occurrence of low antibiotic resistance after acclimatization to the potential inhibitor concentration (Cheng et al. 2020). The dominant bacteria in the anammox system, under combined TC and Zn exposure, were *Candidatus Kuenenia* sp. However, as the concentration of the inhibitors was increased, a dose-dependent decrease in relative bacterial abundance was observed. In contrast, when *Caldilinea* were exposed to an increasing concentration of the inhibitors, a significant increase in its relative abundance was observed. Together, these data suggest that *Caldilinea* is a potentially antibiotic-resistant bacteria (Fan et al. 2019).

The long-term exposure of anammox bacteria to ERY resulted in a decrease in bacterial abundance and the deterioration of nitrogen removal performance in the up-flow anaerobic biological filter (UAF) bioreactor (Zhang et al. 2019b). The most dominant genus in the anammox sludge was *Candidatus Kuenenia* sp*.*, whose relative abundance decreased from 22.04% to 3.60% when exposed to 100 mg L^−1^ of ERY. Without antibiotic stress, the relative abundance recovered to 12.47% after 29 days, however, its abundance could not be rescued to its initial value. Similar changes were observed for another major species of anammox bacteria in the reactor, mainly *Candidatus Brocadia* sp., whose abundance decreased from the initial level of 0.45% to well below detection threshold during exposure to 100 mg L^−1^ of ERY and then increased to 0.01% during recovery phase (Zhang et al. 2019b). Zhang et al. (2019a) indicated that exposure to 1 µg L^−1^ of NOR and ERY has a slight impact on the abundance of the anammox bacteria in the system, which perhaps was due to a very low concentration of antibiotics. In agreement with the study above, the dominant bacteria were identified as *Candidatus Kuenenia* sp. Further, the analysis of the anammox bacterial community abundance showed that the relative abundance of *Candidatus Kuenenia* sp. decreases from initial level 4.31% (NOR) and 5.0% (ERY) to 1.87% and 4.99%, respectively, after 30 days of incubation with these antibiotics.

In a recent study, the inhibitory effect of SDM on anammox bacteria and the abundance of *Candidatus Brocadia* sp. were assessed. Du et al. (2018) found that exposure to SDM facilitated a decrease in relative bacterial abundance from 2.57% to 0.39%. However, the abundance of *Planctomycetes* first increased to 6.76% when exposed to 3 mg L^−1^ and then continuously decreased to 1.52% at a dose of 9 mg L^−1^. Comparatively, the abundance of *Planctomycetes* steadily increased to 28.61% at a dose of 7 mg L^−1^ of SM and dropped rapidly to 11.42% at 9 mg L^−1^. Together, these data show that *Plactomycetes* and *Proteobacteria* are the main groups of bacteria present in the anammox systems. Further, these species have been shown to tolerate a low concentration of antibiotics. Moreover, *Candidatus Kuenenia* sp*.* belonging to the *Planctomycetes* phylum seems to be the most resistant bacteria of all anammox bacteria assessed.

### Mechanism of anammox bacteria inhibition by antibiotics

The mechanisms of action of antibiotics can be divided into two basic categories: bactericidal drugs, which kill bacteria with high efficiency (> 99.9%), and bacteriostatic drugs, which inhibit bacterial growth (Kohanski et al. 2007). Antibiotics can enter through the transporters located in the cell membrane or may connect with receptors on the surface cell (Lu et al. 2021). It is understood that there are four inhibition or bactericidal mechanisms by which antibiotics act on the bacterial cell: (i) inhibition of cell wall synthesis; (ii) interaction with a cell membrane; (iii) inhibition of protein synthesis; (iv) inhibition of the transcription and replication of DNA (Zhang et al. 2019b). Further, the exact mechanism of bacteria inhibition, or killing, is dependent on the antibiotic concentration used. For example, antibiotics belonging to the TCs and CAP group interfere with the synthesis of bacterial mRNA by binding to the subunit of the ribosomes. TCs bind with the small subunit and CAP bind with the large ribosomal subunit (Nguyen et al. 2014). β-lactam antibiotics, such as penicillin inhibit the synthesis of the cell wall through the interaction with the penicillin-binding proteins and glycopeptides that associate with peptidoglycan of cell wall, inducing cell death. Antibiotics belonging to macrolide and aminoglycoside class can easily enter to cytoplasm and then produce intermediates D-glucose and L-streptose which can be further decomposed in the tricarboxylic acid cycle (Lu et al. 2021).

Anammox bacteria are characterized as slow-growth microorganisms. Furthermore, Levin-Reisman et al. (2017) indicated that slow-growing bacteria are more prone to develop antibiotic resistance than fast-growing bacteria. For instance, Liu et al. (2018) showed that slow-growing bacteria involved in the nitrification process are more likely to accept tetracycline resistance genes under trace TC exposure. It could be suspected that bacterial slow growth predisposes them to such adaptation. Zhang et al. (2019c) theorized that, due to the presence of OTC and SMX, there are four possible mechanisms of anammox bacteria that can be used to avoid the deleterious effects of antibiotic exposure: (i) produce protective layer of EPSs, (ii) aggregation of dominant microbes as a potential resistance species, (iii) regulation of the functional genes by dominant microbes as the adaptation effect to the external disturbance, (iv) increasing tolerance of anammox bacteria to SMX and OTC by the increased relative abundance of antibiotic resistance bacteria and ARGs transfer (Zhang et al. 2019c).

Analysis of the ARGs responsible for the acquired antibiotic resistance within anammox systems has highlighted the efflux pump as the most common resistance mechanism against OTC (Shi et al. 2017). Moreover, OTC stress may induce cell lysis (Yang et al. 2013). Sguanci et al. (2017) compared the efficacy of ENR and DOX on the anammox system. In their study, they showed that ENR can inhibit anammox bacteria at a faster rate than DOX. They attributed this effect to intracellular tetracycline and fluoroquinolone transport which may be facilitated by channel-forming proteins (Nikaido and Thanassi 1993). Further, channel-forming proteins genes are common in anammox bacteria (Kartal et al. 2011).

Additionally, ERY inhibited anammox bacteria by binding to the 23S rRNA molecule in the 50S ribosomal subunit, thereby inhibiting the translocation of peptidyl-tRNA and disrupting protein synthesis (Alighardashi et al. 2009; Zhang et al. 2019b). Recent research has shown that a low dose exposure of anammox bacteria to ERY (1 µg L^−1^) can induce the expression of the two ARGs, *ermB* and *mphA*. The presence of *ermB* and *mphA* facilitates obtaining an antibiotic tolerant phenotype. Further, the authors reported that, at similar trace levels of NOR (1 µg L^−1^), there was no protective effect induced (Zhang et al. 2019a). It is understood that *ermB* induces antibiotic tolerance by enhancing the production of polysaccharides on the cell membrane, thus reducing the antibiotics’ ability to penetrate the cell (Zhu et al. 2013). Moreover, *mphA* induces chemical modifications on macrolide antibiotics thereby inducing the degradation of antibiotic or replacing the active group via the expression of synthetic oxidoreductase (Guo et al. 2015, Zhang et al. 2019a). CAP belongs to the class of antibiotics which are effective against Gram-negative bacteria. However, due to the fact that antibiotics are selectively toxic, it can also inhibit prokaryotic and eukaryotic cells alike. CAPs mode of action is achieved through the inhibition of peptide bond synthesis during protein synthesis (Dapena-Mora et al. 2007).

Theoretically, anammox bacteria should not be sensitive to antibiotics which affect the synthesis of the cell wall such as β-lactam antibiotics as many of these species have peptidoglycan-free cell walls (Cayrou et al. 2010; Hu et al. 2013; Zhang et al. 2019b). AMX is a broad-spectrum β-lactam antibiotic belonging to the aminopenicillin family which inhibits cell wall synthesis by interfering with the cross-linking of peptidoglycan (Hu et al. 2013). Sulfonamides act by inhibiting the synthesis of nucleic acids (Lotti et al. 2012b). FF is a widely used chloramphenicol-type antibiotic, which inhibits transpeptidase and disrupts the functional ability of the 50S ribosomal subunit thereby inhibiting protein synthesis (Ding et al. 2015).

An interesting phenomenon was observed during the combined effect of azithromycin, norfloxacin, sulfamethoxazole and trimethoprim on aerobic granules. Bacteria were distributed in the external and intermediate layer of the granule. Further, this external layer was compact, and bacteria were arranged perpendicularly like a protective barrier. This compact layer was likely formed by the accumulation of dead cells (Rodriguez-Sanchez et al. 2017).

### The influence of antibiotics on the antibiotic resistance genes presented in anammox system

Antibiotic resistance is recognized as a global threat to human and animal health, with many bacterial species, including anammox bacteria, having developed some form of resistance toward antibiotics (Felis et al. 2019). One of the main risks for the presence of antibiotics in wastewater is the spread of ARGs (Kumar et al. 2019; Felis et al. 2019). Specifically, the presence of antibiotics in wastewater has been identified as an important factor in the selective adaption of resistance genes in many bacterial species (Zhang et al. 2019b; Shi et al. 2017; Li et al. 2016). Moreover, it is suggested that slow-growing bacteria, as anammox, could contribute to the enrichment of antibiotic resistance genes due to development of antibiotic tolerance (Fu et al. 2020; Langbehn et al. 2021). It is claimed that increasing abundance of ARGs can contribute to proper nitrogen removal, as the bacterial community gains resistance to the exposed antibiotic (Langbehn et al. 2021), which seems to be advantage to nitrogen removal. Moreover, it has been proven that many bacteria involved in nitrogen removal from wastewater or occurring in denitrifying systems, such as *Nitrospira*, *Ignavibacterium*, and *Comamonas* play an important role as ARG-carrying bacteria. The role of anammox bacteria as hotspot of antibiotics resistance genes was investigated by Fan et al. (2021). The authors presented positive correlation between *Candidatus Kuenenia* and *sul1* (gene encoding resistance for sulfonamides). On the other hand, network analysis has shown that *Candidatus Kuenenia* was predicted to be a host of *sul1* and e*rmF* under ETS (erythromycin) + SMZ stress, while under single antibiotics stress both genes were independent, which suggest that potential host of ARGs may depended on different antibiotics stress. Zhang et al. (2020) found strong interaction between *intI1* and *ereA* in anammox system under spiramycin (SPM) exposure. In fact, strong correlation between *intI1* and *ereA* has been previously reported (Thungapathra et al. 2002; Liu et al. 2014) indicating that anammox bacteria belonging to *Planctomycetes* can be the host of ARGs in nitrogen removal systems.

Zhang et al. (2019c) investigated the presence and changes in the absolute abundance of ARGs such as *tetX*, *tetC*, *tetG*, *tetM*, *sul1*, *sul2* in the anammox biomass conducted in UASB reactor under OTC, SMX and OTC + SMX antibiotic pressure. These genes can be divided into three groups according to the different resistance mechanisms: efflux pump (*tetC*, *tetG*), ribosome protection (*tetM*) and enzymatic inactivation (*tetX, sul1*, *sul2*). In their study, the highest abundance was presented by *tetX* which was more than one order of magnitude greater than that of *tetG*, *tetM*, *intI1*, *sul1*, *sul2*. Additionally, *tetC* was identified to have the lowest abundance (Zhang et al. 2019d). Similarly, Zhang et al. (2019d) reported that, under OTC drug pressure, the highest absolute abundance of ARGs were *tetX* and the lowest for *tetC*. Further, the total number of ARGs increased after the acclimation of anammox sludge to the long-term exposure of OTC. Typical tetracycline resistance genes: *tetA*, *tetB*, *tetC* and *tetX* were also detected by Shi et al. (2017). In that study, the relative abundance of *tetA*, *tetB*, *tetC* increased after the addition of OTC (1 mg L^−1^) and the highest peak was reached on day 52, where the concentration of OTC was 2 mg L^−1^. Overall, the relative abundance of the ARGs assessed in descending order was as follows: *tetA* > *tetX* > *tetC* > *tetB*. After 1 month of exposure to NOR and ERY (1 µg L^−1^), two resistance genes (*ermB*, *mphA*) were detected at a low abundance, both of which target ERY (Zhang et al. 2019a). The relative expression of *ermB* increased from 2.08 × 10^−4^ to 3.53 × 10^−4^%. Additionally, *mphA* increased from 4.48 × 10^−5^ to 5.00 × 10^−4^%. It is worth noting that during this experiment the presence of NOR caused a significant decrease in nitrogen removal efficiency. Further, ERY exposure slightly impacted the anammox process, highlighting the role of ERY-ARGs on anammox bacteria in the induction of protection against this antibiotic.

### Recovery of anammox process after antibiotics inhibition

Depending on the type of the substance and concentration of the antibiotic tested, the negative effect of antibiotics on anammox performance can be either reversible or irreversible (Gonzalez-Martinez et al. 2018). Fernández et al. (2009) reported that, after incubation with 20 mg L^−1^ of CAP, the anammox process can recover up to 59% of its initial level after 2 months. However, exposure to tetracycline hydrochloride (100–1000 mg L^−1^) induced a complete deactivation of the biomass. In this instance, the recovery of the anammox process would require a fresh inoculation of anammox bacteria. Other authors have suggested that the long-term acclimatization of anammox bacteria to the conditions in the reactor, in which the process is conducted, is useful for the recovery of stable anammox performance (Zhang et al. 2015; Rodriguez-Sanchez et al. 2017).

Two strategies of the recovery of anammox performance after OTC shock were assessed by Yang et al. (2013): (i) addition of low concentration of antibiotics within a long recovery period, (ii) no OTC addition combined with sludge addition. Despite the use of strategy (i) comprising of low concentration exposure of OTC (6 mg L^−1^) and long recovery period (36 days), it would be difficult to recover the anammox process after high level of OTC used (50 mg L^−1^). A more useful strategy to facilitate the recovery if the anammox performance would be the addition of fresh anammox sludge. The increase in volatile suspended solid (VSS) by approximately 3.3 g resulted in an increase in SAA from a 0.8 mg TN g^−1^ VSS^−1^ to 13.7 mg TN g^−1^ VSS^−1^. The addition of sludge is a powerful method to recover the nitrogen removal capability which is lost due to the action of contaminants. This strategy was also confirmed by Tang et al. (2011) and Yang and Jin (2012). Zhang et al. (2019d) investigated two possible mechanisms of the anammox recovery after OTC exposure: addition of fresh sludge without discharge from this reactor throughout the experiment and addition of fresh anammox sludge with discharge the same volume of sludge. Obtained results have shown that exchange of biomass was superior to addition biomass in recovering the anammox performance after OTC exposure. Moreover, they reported that recovery mechanism performance aided with BA is connected with the combined contribution of functional gene regulation, the presence of efflux pumping ARGs and the self-defense role of EPSs (Zhang et al. 2019d). Yao et al. (2018) suggested that the addition of anammox sludge can stimulate the recovery process as a result of quorum sensing (QS). However, this hypothesis requires further validatory research. Taken together, bio-augmentation, through the addition of sludge, can be a useful method to mitigate the inhibitory effect of OTC, due to gradually acclimation of the microbial communities (Jin et al. 2014). Moreover, Jin et al. (2014) suggested that there are two major parameters influencing the proper recovery process: bio-augmentation time (BAT) and bio-augmentation dosage (BAD).

The negative effect of antibiotics on the anammox process can be also mitigated by adsorption and biodegradation of antibiotics by anammox bacteria (Alvarino et al. 2015). Zhang et al. (2014) suggested that recovery was mainly attributed to biodegradation of residual OTC under anaerobic conditions. However, there is lack of information about possible biodegradation mechanism. Degradation of sulfadiazine; sulfamethazine, and sulfamethoxazole by nitrogen cycle bacteria was speculated by Langbehn et al. (2021). The suggested degradation pathway was deamination catalyzed by deaminase, hydroxylation by ammonia monooxygenase, and nitration by hy-droxylamine dehydrogenase. Therefore, several enzymes are able to catalyze antibiotic degradation during the biological nitrogen removal. However, it depends on the antibiotic molecular properties and cannot be unified to all antibiotics (Langbehn et al. 2021). In case of OTC, no sorption and volatilization would be expected (Carucci et al. 2006; Sponza and Çelebi 2012). On the other hand, some studies have reported good sorption capacity of tetracyclines antibiotics onto activated sludge flocs surface (Felis et al. 2019), which caused the accumulation of this substance in sludge. Moreover, trace concentration of such antibiotics as SM, sulfamerazine and, sulfadiazine can be adsorbed by hydrophobic regions of EPSs, making anammox process easy to recover, because these antibiotics don’t affect the bacteria (Hou et al. 2016). However, this led to accumulation of these antibiotics in sludge. Unfortunately, there is a lack of knowledge describing the impact of antibiotics accumulation on the anammox process and process recovery. Zhang et al. (2013) concluded that the changes in the pH value, stoichiometric ratios, SAA and properties of the anammox granules could be useful indicators for the degree of anammox recovery. Moreover, the recovery velocity, recovery time and degree of recovery are important indices for evaluating the recoverability of the anammox performance within the system (Cai et al. 2009; Zhang et al. 2014).

### Influence of antibiotics on the anammox biomass properties

#### Production of EPSs

EPSs play an important role in anammox sludge aggregation. Their physicochemical properties such as surface charge, settling properties, dewatering properties, flocculation and adsorption ability and location outside the cell make them an important part for maintaining microbial aggregate’s structure and function (Sheng et al. 2010; Zhang et al. 2014). Moreover, EPSs can affect wastewater treatment capacity and are produced in response to stressors such as the presence of antibiotics (Zhang et al. 2014, 2018a, 2019c). The main components of EPSs are proteins and polysaccharides which can influence the bacterial surface charge, hydrophobicity and sorption capacity. These proteins can provide adsorption abilities due to the hydrophobic region which include functional groups such as carboxyl, amine and hydroxyl group (Sheng et al. 2010). Therefore, EPSs can adsorb tetracycline antibiotics and sulfamethazine, sulfamerazine and sulfadiazine (Hou et al. 2016; Zhang et al. 2019c).

Zhang et al. (2018b) reported that increasing the concentration of NOR from 0.001 to 100 mg L^−1^ facilitated a drastic decrease in detectable protein from 74.06 mg g^−1^ SS to 24.04 mg g^−1^ SS at 0.001 mg NOR L^−1^ which gradually increased to 198.93 mg g^−1^ SS in 50 mg NOR L^−1^. However, at 100 mg L^−1^ NOR, the decrease of detectable proteins was observed again. Like the changes in protein abundance, the dynamics of detectable polysaccharides elicited a similar trend in response to antibiotics pressure. These studies show that as the anammox bacteria begin to acclimatize to the presence of NOR, the anammox biofilm secreted more EPSs, mainly proteins which had a significant impact on antibiotic protection (Zhang et al. 2018b). However, at 100 mg L^−1^ of NOR, the concentration of antibiotic exceeded the resistance capacity of the anammox biofilm, and the process was inhibited, as measured by a decrease in EPSs. These results agree with the finding of Zhang et al. (2019b) who indicated that at a concentration of 0.001–50 mg L^−1^, ERY induced a significant increase in EPSs production. However, at a concentration of 100 mg L^−1^, the secretion of EPSs was reduced. Zhang et al. (2019c) observed that, during a gradual increase in OTC and SMX (0.1–1 mg L^−1^) concentration, the EPSs content initially decreased, then increased at 0.5 mg L^−1^ and then decreased further with 1 mg L^−1^. It is worth noting that the final EPSs content was higher than the initial measurement (Zhang et al. 2018b, 2019a, b, c, d). Du et al. (2018) observed that SDM and SM above 5 mg L^−1^ caused a decrease in EPSs secretion, however, at concentrations lower than 3 mg L^−1^ the opposite effect was observed. Taken together, these data show that EPSs serves as a protective barrier to delay the penetration of hazardous agents into the cell, however, EPS-defense can be ablated by antibiotic overload (Zhang et al. 2015, 2016).

#### Heme c synthesis

Heme is an important part of the anammox key enzymes such as hydrazine synthesis (*hzs*), hydroxylamine oxidoreductase (*hao*) and hydrazine oxidase (*hzo*) which are involved in energy metabolic pathways (Jetten et al. 2009; Zhang et al. 2014). Moreover, the content of heme c may relate directly to anammox activity (Chen et al., 2013; Sabine Marie et al. 2015). Zhang et al. (2014) investigated influence of OTC (518 mg L^−1^) on the anammox process under shock exposure while monitoring the changes of heme c in pre-, during- and post-shock period. The content of heme c before the shock period was 0.17 ± 0.001 µmol g^−1^ VSS. During the shock period, the level of heme c was reduced to 0.14 ± 0.001 µmol g^−1^ VSS. Additionally, after 105 h of recovery time, the heme c content was 0.12 ± 0.001 µmol g^−1^ VSS. A similar trend was observed during a higher OTC concentration (1731 mg L^−1^) shock treatment. Changes in heme c coincided with a decrease in the anammox activity, but the recovery of heme c was delayed. Other studies have indicated that concentrations of OTC and SMX ranging between 0.1 and 1 mg L^−1^ elicited a decrease in heme c content in the anammox granules assessed. However, a mixture of these antibiotics caused fluctuations in the heme c content. For instance, the addition of 0.5 mg L^−1^ OTC + SMX increased the heme c content by 8.78%, meanwhile the addition of 1 mg L^−1^ OTC + SMX decreased the heme c content by 70.1%. These results differ in resistance properties to anammox bacteria by separated and mixed antibiotics (Zhang et al. 2019c). An increase in heme c content was also observed by Meng et al. (2019). The authors found that under a low concentration of TC (1, 10 and 100 μg L^−1^) heme c content was increased, whereas exposure to 1000 µg L^−1^ caused a decrease in heme c content. Further, the authors found that FF rapidly reduced the heme c content by 20% from within 3 days, however, when the addition of FF is stopped, heme c returned to baseline (Zhang et al. 2015). It was reported that under OTC exposure, the color of anammox granules were fading (Yang et al. 2013). The red color of anammox sludge is attributed to the presence of heme c (Shi et al. 2017). Thus, the loss of red color is frequently regarded as an indicator of anammox activity (Tang et al. 2011; Yang et al. 2013).

## Conclusion and prospects

Anammox is one of the most promising processes for nitrogen-rich wastewater removal, thus it is important to understand the impact of potential inhibitors present in the wastewater which may affect this process. There is no doubt that antibiotic pollution is a serious threat to the anammox process. Previous studies have shown that they can decrease anammox process activity, change community structures and alter the properties of the anammox biomass. Further, the effect of antibiotics depends on numerous factors such as antibiotic type, concentration or presence of coexisting inhibitors/pollutants (heavy metals, sulfides, nanoparticles). Knowledge of the impact of antibiotic and other co-pollutants on the anammox process is still unclear. Within this context, further research should focus on:

- Extending knowledge on the different antibiotics present in wastewater which may impact the anammox process. Further, the varying cellular structure and metabolism of anammox bacteria relative to other wastewater treatment bacteria, implies that the effect of antibiotics may be different and should be assessed further. However, there are a lot of antibiotics that occur in high concentrations in the aquatic environment. One such example is ciprofloxacin which was found in 6453 ng L^−1^ in raw wastewater. It is likely that, at these concentrations, this antibiotic may constitute a serious risk to activated sludge microorganisms. Moreover, as discussed in this review, some antibiotics such as β-lactams, have been thought to have no effect on anammox processes. However, their negative effect on the anammox system has been experimentally confirmed. Therefore, it is worthwhile to study a broad spectrum of antibiotics on the anammox process to create a holistic understanding of these compounds on this system.
- Extending knowledge about the combined effect of antibiotics and other pollutants which may co-occur in wastewater. As was reported above, antibiotics always occur simultaneously with other contaminants in the aquatic environment, especially in wastewater. Besides the synergistic or antagonistic effect of combined contaminants on the anammox process effectiveness, these contaminants can also affect the spread of ARGs. The presence of antibiotics and heavy metals may stimulate the development of ARGs and heavy metals resistance gene expression, which are located in one of the mobile elements, such as plasmids or transposons. Moreover, a positive correlation between heavy metals stress and the abundance of ARGs was reported (Xu et al. 2017).
- Extending knowledge on the fate of antibiotics in the anammox systems. Antibiotics may undergo various changes in biological systems, i.e., biodegradation, biotransformation and sorption. Sorption is an important aspect in the field of ciprofloxacin (CIP) research. Previous studies have shown that 80% can be adsorbed into sludge (Golet et al. 2003, Michael et al. 2013, Felis et al. 2019). A similar effect was described for TC (Felis et al. 2019). Biodegradation and biotransformation may result in the accumulation of metabolites or incomplete transformation products. Further, the partial metabolites of antibiotics can be more dangerous than antibiotics themselves. For example, amoxicillin-S-oxide is a metabolite of amoxicillin and contain β-lactam ring, which may lead to the development of resistance in bacterial cells (Elizalde-Velázquez et al. 2016). Understanding the fate of antibiotics and their metabolites will be key to establishing efficient anammox systems in the future.
- The effect of antibiotics on combined processes. To improve the efficiency of nitrogen removal from wastewater, the anammox process is often combined with partial nitrification or partial denitrification. Few studies have investigated the effects of antibiotics on coupled processes, where their response to antibiotics may vary. This is a topic on which research should be focused on future studies.
- Previous studies focused mainly on synthetic antibiotic-contaminated wastewater treatment in the anammox process under laboratory condition. Therefore, there is a need to evaluate effect of real antibiotic-contaminated wastewater in the pilot-scale or full-scale systems to allow practical application of the anammox process.
- Studies should be conducted to determine the concentration of antibiotics that occur in wastewater treated by the anammox process. There are many studies in which the anammox process is used to treat wastewater containing antibiotics, but the concentration of antibiotics in the wastewater is not tested. Therefore, it is not possible to evaluate the concentration effect of antibiotics on the anammox process, because the concentration is unknown. This will be essential for both, future process studies and the establishment of efficient anammox systems in the future.

## Data Availability

Not applicable.
